# Phenome-Wide Association Study of Polygenic Risk Score for Alzheimer’s Disease in Electronic Health Records

**DOI:** 10.3389/fnagi.2022.800375

**Published:** 2022-03-15

**Authors:** Mingzhou Fu, UCLA Precision Health Data Discovery Repository Working Group, Anna L. Antonio, UCLA Precision Health ATLAS Working Group, Ruth Jhnson, Timothy S. Chang

**Affiliations:** ^1^Movement Disorders Program, Department of Neurology, David Geffen School of Medicine, University of California, Los Angeles, Los Angeles, CA, United States; ^2^Medical Informatics Home Area, Department of Bioinformatics, University of California, Los Angeles, Los Angeles, CA, United States

**Keywords:** Alzheimer’s disease, polygenic risk score, phenome-wide association study, electronic health record, Mendelian randomization

## Abstract

Alzheimer’s disease (AD) is the most common form of dementia and a growing public health burden in the United States. Significant progress has been made in identifying genetic risk for AD, but limited studies have investigated how AD genetic risk may be associated with other disease conditions in an unbiased fashion. In this study, we conducted a phenome-wide association study (PheWAS) by genetic ancestry groups within a large academic health system using the polygenic risk score (PRS) for AD. PRS was calculated using LDpred2 with genome-wide association study (GWAS) summary statistics. Phenotypes were extracted from electronic health record (EHR) diagnosis codes and mapped to more clinically meaningful phecodes. Logistic regression with Firth’s bias correction was used for PRS phenotype analyses. Mendelian randomization was used to examine causality in significant PheWAS associations. Our results showed a strong association between AD PRS and AD phenotype in European ancestry (OR = 1.26, 95% CI: 1.13, 1.40). Among a total of 1,515 PheWAS tests within the European sample, we observed strong associations of AD PRS with AD and related phenotypes, which include mild cognitive impairment (MCI), memory loss, and dementias. We observed a phenome-wide significant association between AD PRS and gouty arthropathy (OR = 0.90, adjusted *p* = 0.05). Further causal inference tests with Mendelian randomization showed that gout was not causally associated with AD. We concluded that genetic predisposition of AD was negatively associated with gout, but gout was not a causal risk factor for AD. Our study evaluated AD PRS in a real-world EHR setting and provided evidence that AD PRS may help to identify individuals who are genetically at risk of AD and other related phenotypes. We identified non-neurodegenerative diseases associated with AD PRS, which is essential to understand the genetic architecture of AD and potential side effects of drugs targeting genetic risk factors of AD. Together, these findings expand our understanding of AD genetic and clinical risk factors, which provide a framework for continued research in aging with the growing number of real-world EHR linked with genetic data.

## Introduction

Dementia is one of the largest unmet medical needs worldwide. Alzheimer’s disease (AD) is the most prevalent form of dementia, which accounts for 60–70% of the total cases (Alzheimer’s Association, 2021). In the United States, an estimated 6.2 million individuals aged 65 and older are living with AD, which results in an economic cost of $355 billion (Alzheimer’s Association, 2021). Multiple factors, both genetic and environmental, are associated with AD (Xu et al., 2015). Genome-wide association studies (GWASs) have identified multiple common variants, which together contribute to 7.1% of the risk for AD (Kunkle et al., 2019). Well-established genetic risk factors include the ε4 allele of the apolipoprotein E (*APOE*) gene, the five repeat allele of very low-density lipoprotein receptor (*VLDL-R*) gene, and deletion in exon 18 of the α2 macroglobulin (*A2M*) gene (Tilley et al., 1998). Environmental factors, such as air pollution, dyslipidemia, and type 2 diabetes, are also associated with higher risk of AD (Tsuno and Homma, 2009; Fu et al., 2021; Ware et al., 2021). Given the large public health burden, determining the relationship between AD genetic risk and other disease conditions can improve our understanding of the genetic architecture of AD and disease conditions that may be the risk factors for AD.

A phenome-wide association study (PheWAS) can identify the shared genetic etiology between AD and other diseases. A PheWAS is considered a genotype-to-phenotype approach where multiple phenotypes are tested for association with one genetic loci (Hebbring, 2014). As a way of exploring gene-disease associations, PheWAS has been used by investigators with extensively phenotyped cohorts such as large biobanks (Bycroft et al., 2018) and electronic health record (EHR) systems (Denny et al., 2013).

To define phenotypes, PheWASs use computable phenotypes derived from EHR databases. Standard PheWASs have primarily focused on correlating single-nucleotide polymorphisms (SNPs) to a spectrum of phenotypes, which may result in limited power due to the small effect size of each SNP (Fritsche et al., 2018). A polygenic risk score (PRS) is a summary score calculated by aggregating the risk carried by multiple genetic variants, weighted by their effect sizes from a GWAS (Escott-Price et al., 2015). As a measurement of genetic liability to a trait, the PRS has shown promise in predicting human complex traits and diseases and may facilitate early detection, risk stratification, and prevention of common complex diseases (Chatterjee et al., 2016). For instance, one study reported an area under the curve (AUC) of 0.57 using *APOE* region only to predict AD (Tosto et al., 2017), whereas another study reported an AUC of 0.84 with an AD PRS using more than 2,00,000 variants including *APOE* (Escott-Price et al., 2017).

Because a PheWAS identifies multiple phenotypes associated with AD genetic risk, it is possible that these PheWAS significant phenotypes are the causal risk factors for AD. For example, AD genetic risk may lead to a PheWAS significant phenotype, which may lead to AD. Mendelian randomization (MR) is a method using genetic variants as the instrumental variables to assess causality between two phenotypes known as the exposure and the outcome. It is analogous to a randomized control trial where individuals are randomized to carry genetic variants that may modify the risk of an exposure. Since genetic variants are fixed at conception, preceding the onset of health disorders and environmental exposures, MR can overcome many drawbacks of observational studies, such as confounding and reverse causation (Smith and Ebrahim, 2003).

Our study is the first to perform a comprehensive PheWAS from AD PRS in an academic health center EHR with different ancestry populations. We first constructed AD PRS based on the largest AD GWAS (Kunkle et al., 2019). Then, we linked EHR information with genotypic data to explore phenotype associations of AD genetic risk. When a PRS-based PheWAS led to the association with other phenotypes (e.g., gout), we performed MR to evaluate their causal relationships.

## Subjects and Methods

### University of California, Los Angeles ATLAS Cohort

Participants were recruited through University of California, Los Angeles (UCLA) Health System. Written informed consent was obtained from the participants for the study of remnant biosamples in the UCLA ATLAS Precision Health Biobank (Chang et al., 2021; GenomicsDB, 2021; Johnson et al., 2021). Genetic data obtained from remnant biosamples as described below were linked to the deidentified EHR from the UCLA Health System known as the UCLA Data Discovery Repository (DDR), developed under the auspices of the UCLA Health Office of Health Informatics Analytics and the UCLA Institute of Precision Health. This study was considered human subject research exempt because all genetic and EHRs were deidentified (UCLA IRB# 21-000435).

### Data Preprocessing

#### Genotyping and Sample Quality Control

Genotype collection, quality control, processing, and imputation were performed by the UCLA ATLAS Precision Health Biobank (GenomicsDB, 2021; Johnson et al., 2021). Briefly, DNA was extracted from participant blood samples and genotyped on a custom Illumina Global Screening Array that included a standard GWAS backbone and an additional set of pathogenic variants selected from ClinVar (Landrum et al., 2018). Preprocessing of the genotyped data includes removing contaminated samples, unmapped SNPs, high missing rate samples, high missing rate SNPs, duplicates, and performing strand flip (PLINK v.1.90) (Chang et al., 2015; Johnson et al., 2021). After performing array-level genotype quality control, genotypes were imputed from the Michigan Imputation Server (2021). After filtered by *R^2^* > 0.90 and minor allele frequency (MAF) > 0.01, 8,048,268 polymorphic variants and 30,118 participants remained.

Population stratification, defined as the presence of systematic allele frequency differences between populations, can distort the true effect estimates between genetic variants and disease (Price et al., 2006). To adjust for population stratification, we conducted all analyses within samples of the same genetic ancestry group. We inferred samples’ genetic ancestry by projecting all genotyped samples into the principal components (PCs) space of the 1,000 Genome Project (phase 3) (Internationalgenome1000, 2021) reference panel using the R package “bigsnpr” (Privé et al., 2018). We limited the principal component analysis (PCA) to variants that were shared between the 1,000 Genome reference and the UCLA ATLAS data, had a MAF > 0.01, and remained after linkage disequilibrium (LD) clumping (*R^2^* > 0.2, prioritizing variants by higher minor allele counts). PCs were stored and used for further association tests. Genetic ancestry of each sample was inferred using k-nearest neighbor (k-NN) (Altman, 1992) (multiclass classification) with the first 20 PCs of the genotyped data. Genetic ancestry classes were assigned to European, African, American, East Asian, or South Asian ancestry. We compared patients’ inferred genetic ancestry with self-reported race or ethnicity, and results are shown in Supplementary Table 1.

#### Phenotype Generation

International Classification of Disease (ICD) codes are standard diagnosis codes used in the EHR. ICD codes are arranged hierarchically to describe diseases and syndromes. It has fine granularity but are considered too detailed to represent clinically meaningful phenotypes and to replicate known genetic associations (Wei et al., 2017). Instead, we used phecodes in our study to reduce complexity of phenotypes in the EHR. Phecodes are defined as a combination of ICD codes and have been validated by experts to better represent clinical disease phenotypes (Denny et al., 2010). As such, this improves power to detect an association by increasing the number of cases and reducing multiple hypothesis testing. We extracted the diagnosis data (ICD-9/10 codes) from all types of encounters (including appointment, hospital encounter, office visit, history, telephone, patient message, orders, transcribed document, scanned document, billing encounter, refill, letter, laboratory visit, health maintenance letter, procedure pass, ancillary orders, historical scanned document, and ancillary procedure) from the UCLA EHR and mapped the ICD codes to phecodes using the R package “PheWAS” (Carroll et al., 2014). Cases for a given PheWAS code were defined if an individual had at least one assignment of that phecode in their longitudinal records. The remaining individuals that did not have phecodes from exclusion criteria previously defined (Carroll et al., 2014) were considered as control subjects. In each ancestry sample, we only tested phenotypes with ≥ 50 cases and ≥ 50 controls to increase statistical power in the PheWAS analyses. A total of 1,515 case–control studies were generated for further analyses.

### Construction of Alzheimer’s Disease Polygenic Risk Score

To construct the AD PRS, we used the summary statistics of a late-onset AD GWAS conducted by Kunkle et al. (2019) in which included 21,982 cases and 41,944 controls (N SNP = 11,480,632). Variant positions were converted to GRCh38 using variant IDs from dbSNP build 151 (UCSC Genome Browsers) (Karolchik et al., 2004). The set of SNPs that overlapped between GWAS summary statistics and ATLAS genotyped data was retained for PRS construction. We also restricted our analyses to only the HapMap3 SNPs and removed outliers (SNPs) from the summary statistics as recommended by Privé et al. (2020). A total of 953,397 SNPs passed the above quality control steps and were used for PRS construction. We then used LDpred2 to build the AD PRS (Privé et al., 2020). For the first step of LDpred2, we used a reference dataset from 1,000 Genome (European samples only, *n* = 522) to extract overlapping GWAS hits and estimated pairwise LD using the available allele dosages of the corresponding controls. LDpred2 updated weights (β) based on LD information and then the updated weights were applied to all UCLA ATLAS samples accordingly. The PRS was calculated by the sum of an individual’s risk allele dosages, weighted by risk allele effect sizes. Namely, for subject *j*, the PRS was of the form *PRS*_*j*_ = ∑_*i*_β_*i*_*G*_*ij*_ where β_*i*_ was the updated weight for locus *i*, and *G*_*ij*_ was the measured dosage data from the risk allele on locus *i* in subject *j*. The same methods were applied to construct AD PRS for each ancestry group. Finally, we normalized all PRSs (mean = 0, standard deviation = 1) to a reference population (the 1,000 Genome, European sample).

### Statistical Analysis

To validate the AD PRS, we examined the association between PRS and AD phenotype (phecode = 290.11) using logistic regression. To avoid selection bias introduced by younger, healthier participants, we only selected people aged over 65 without AD as our controls (vs. AD cases). We first determined the PRS quartiles within each ancestry sample, categorized all samples according to these PRS quartiles, and fitted logistic regression adjusting for age, sex, and the first five PCs. We reported area under the receiver operating characteristic (ROC) curve (AUC) (Receiver Operating Characteristic, 2021) and odds ratios (ORs) corresponding to the top vs. the bottom quartile PRS (reference), referred to as PRS OR. We also used continuous PRS as the covariate to increase statistical power.

For our primary PRS PheWAS, we conducted logistic regression for each phenotype, adjusting for age, sex, and the first five PCs. We used Firth’s bias reduction method in logistic regression models to avoid the problem of separation, which is introduced by very small observed value of the outcome that leads to large parameter estimates and standard errors in a binary or categorical outcome logistic regression (Wang, 2014). We applied the false discovery rate (FDR) *p*-value correction to adjust for multiple testing (Korthauer et al., 2019). The results were presented as ORs and raw or adjusted *p*-values.

For significant PheWAS hits on AD PRS, we first reexamined their associations with AD PRS within non-AD controls only. We also tested their relationship with AD phenotype in our sample using logistic regression and one-sample MR. Confounders used for model adjustments were health conditions that were associated with both phenotypes. The conceptual directed acyclic graph and MR assumptions are shown in Supplementary Figure 1. For one-sample MR, sequential probit models were used to calculate the causal effect controlling for confounders at each step (Davies et al., 2018). We also used two-sample MR, which uses large GWAS summary statistics (Hartwig et al., 2016), to test the robustness of our one-sample MR results. In two-sample MR, identified SNPs at significance thresholds (liberal: *P* < 1E-06; conservative: *P* < 5E-08) were clumped for independence using PLINK clumping (*R*^2^ ≤ 0.001, window size = 10,000 kb) within a European reference panel, where SNPs with the lowest *p*-value were retained. We applied multiple robust methods in our study including inverse variance weighted (IVW, with multiplicative random effects model), MR-Egger, weighted median, and weighted mode. Beta coefficient, standard error, and *p*-value were reported for each method. Finally, we performed multiple sensitivity analyses to test whether those MR assumptions were met. F-statistics was used to check instrumental variable strength, with > 10, which indicates a sufficiently strong instrument (Burgess et al., 2011). Cochran’s *Q*-test, MR-Egger intercept, and MR-PRESSO global test were used to examine the existence of horizontal pleiotropy and outliers (Bowden et al., 2018; Verbanck et al., 2018). Additionally, I^2^ statistics was calculated as a measure of heterogeneity between causal estimates, with a low I^2^ which indicates estimates biased toward the null (Bowden et al., 2016).

All analyses were carried out separately for different genetic ancestries. If not stated otherwise, analyses were performed using R version 4.1.0 (R: The R Project For Statistical Computing, 2019).

## Results

The study cohort included 30,118 genotyped samples with EHR data (see summary characteristics of the cohort in Table 1). The study cohort contained 54.6% women and the median age was 61 years. Of these samples, 0.92% had a diagnosis of AD. Compared to non-European genetic ancestry samples, the European sample was older and had a lower AD PRS. The African and South Asian samples had a higher proportions of AD cases.

**TABLE 1:** Demographics and clinical characteristics of UCLA ATLAS sample.*^a^*

		Genetic ancestry sample					
	All sample	European	African	American	East Asian	South Asian	Overall *P*-value*^b^*
Characteristic	*n* = 30,118	*n* = 19,934	*n* = 1,663	*n* = 4,991	*n* = 2,982	*n* = 548	
Females, N (%)	16,434 (54.6%)	10,288 (51.6%)	1,027 (61.8%)	3,004 (60.2%)	1,816 (60.9%)	299 (54.6%)	< 0.001*
Age (years), Median [25th;75th]	61.0 [45.0;72.0]	63.0 [48.0;73.0]	60.0 [46.0;71.0]	53.0 [39.0;66.0]	57.0 [42.0;70.0]	49.0 [38.0;66.0]	< 0.001*
Encounters per participant, Median [25th;75th]^c^	59.0 [25.0;119]	59.0 [26.0;119]	73.0 [29.0;152]	55.0 [22.0;119]	54.0 [25.0;105]	49.0 [23.0;106]	< 0.001*
Unique diagnosis per participant, Median [25th;75th]	59.0 [32.0;103]	60.0 [33.0;103]	71.0 [38.0;125]	59.0 [29.0;107]	50.0 [28.0;88.0]	51.0 [28.0;83.0]	< 0.001*
Timespan of records (years), Median [25th;75th]	6.00 [3.10;8.10]	6.20 [3.30;8.10]	6.50 [3.30;8.20]	5.20 [2.50;7.90]	5.80 [3.00;8.00]	5.30 [2.90;7.80]	< 0.001*
PRS for Alzheimer’s disease, Mean (SD)	0.46 (1.64)	0.16 (1.43)	3.63 (1.58)	0.23 (1.40)	1.07 (1.34)	0.19 (1.39)	< 0.001*
Alzheimer’s disease case count, N (%)	241 (0.92%)	168 (0.97%)	15 (1.06%)	34 (0.78%)	17 (0.63%)	7 (1.42%)	0.21

*PRS, polygenic risk score; SD, standard deviation.*

*^a^All the statistics were calculated based on non-missing data for each variable.*

*^b^Depending whether the row variable is considered as continuous normal distributed (1), continuous non-normal distributed (2) or categorical (3), the following descriptives and tests are performed: 1- mean, sd, and ANOVA; 2- median, 1st and 3rd quartiles, and Kruskal–Wallis test; (3), absolute and relative frequencies and chi-squared or exact Fisher’s test when the expected frequency is less than 5 in some cells from chi-square test for categorical variables as appropriate, interpreted as differences between groups.*

^c^All types of encounters extracted from the EHRs were included, see details in section “Subjects and Methods.” *significant test statistics (p < 0.05).

### Validation of Alzheimer’s Disease Polygenic Risk Score

To validate the construction of AD PRS, we determined the association between AD PRS and AD in our UCLA ATLAS sample by ancestry (Table 2). AD PRS was positively associated with AD phenotype in the European and East Asian ancestry sample. After adjusting for age, sex, and first five PCs, European participants falling in the top quartile of AD PRS (>0.954) were associated with 1.81 (95%CI: 1.18, 2.82) times higher odds of AD relative to the bottom quartile (≤ -0.854); the odds were higher in East Asian participants, though with a wider confidence interval (OR = 5.11, 95% CI: 1.09, 37.77). A one standard deviation unit increase in AD PRS was associated with 1.26 (95% CI: 1.13, 1.40) times higher odds of AD in European ancestry and 1.88 (95% CI: 1.22, 2.98) times higher in East Asian ancestry. For European ancestry, the AUC for AD PRS alone to predict AD in the logistic regression model was 0.58 (95% CI: 0.53, 0.63) and increased to 0.79 (95% CI: 0.74, 0.83) with covariates including age, sex, and first five PCs. However, no association was observed between AD PRS and AD in other ancestry groups. Taken results together, the AD PRS built using GWAS summary statistic from European ancestry individuals (Kunkle et al., 2019) was confirmed to be a valid instrument for further analyses in the European and East Asian ancestry but should be used with caution for other ancestry samples.

**TABLE 2:** Associations between AD PRS and AD, UCLA ATLAS sample, by genetic ancestry.*^a^*

	Categorical (top vs. bottom quartile) PRS			Continuous PRS				
	N*^b^*	Odds Ratio (95%CI)		N*^b^*	Odds Ratio (95%CI)		AUC: PRS alone (95%CI)	
European	3,829	1.81	(1.18, 2.82)	7,620	1.26	(1.13, 1.40)	0.58	(0.53, 0.63)
African	262	0.91	(0.11, 7.79)	521	0.95	(0.63, 1.41)	0.55	(0.40, 0.70)
American	544	0.62	(0.21, 1.72)	1,084	0.97	(0.74, 1.26)	0.50	(0.40, 0.61)
East Asian	448	5.11	(1.09, 37.77)	905	1.88	(1.22, 2.98)	0.66	(0.53, 0.79)
South Asian	65	0.46	(0.01, 7.46)	123	0.80	(0.40, 1.51)	0.60	(0.31, 0.88)

*AD, Alzheimer’s Disease; CI, confidence interval; PRS, polygenic risk score.*

*^a^All the values were based on results from multivariable logistic regression analyses in each sample, in which “no AD” was used as the reference group. ORs were reported from the models which further adjusted for age, sex, and first five PC sets. AUCs were reported from the models with PRS alone.*

*^b^This number include both AD cases and controls.*

### Alzheimer’s Disease Polygenic Risk Score Phenome-Wide Association Study

**FIGURE 1:**
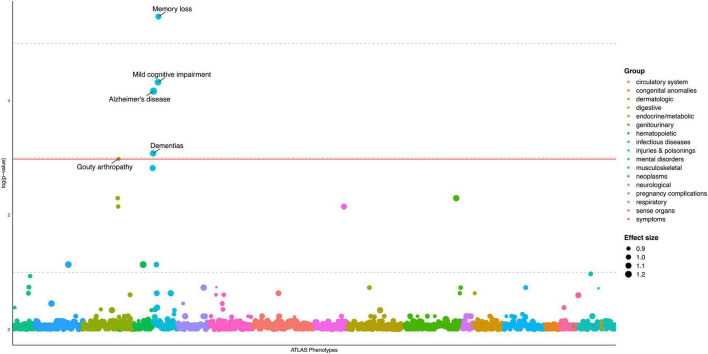
PheWAS plot for Alzheimer’s disease polygenic risk score, European ancestry sample (*N* = 19,934). 1515 traits (number of cases/controls ≥ 50) are grouped into 17 color-coded categories as shown on the horizontal axis; the *p*-values for testing the associations of PRS with the traits were adjusted by FDR and transformed to minus natural logarithms, shown on the vertical axis. The size of the dot refers to effect size (OR) of AD PRS on traits. All values were based on results from multivariable logistic regression analyses, in which “no disease/symptom” was used as the reference group, adjusted for age, sex, and first five PCs. The solid horizontal line for adjusted *p* = 0.05 cutoff.

We evaluated AD PRS across 1515 EHR-derived phenotypes with at least 50 case and control subjects in the European sample as our primary analyses (Supplementary Table 2A). Through a PheWAS plot, we present -ln(FDR corrected *p*-values) corresponding to each of the 1,515 association tests for H_0_: β_*PRS*_ = 0 (Figure 1). After FDR *p*-value correction, we found strongest associations of AD PRS with the AD and related phenotypes, which include mild cognitive impairment (MCI) (OR = 1.18, FDR = 0.013), memory loss (OR = 1.10, FDR = 0.004), and dementias (OR = 1.14, FDR = 0.046) (Table 3). We observed a borderline association between AD PRS and delirium dementia and amnestic and other cognitive disorders (OR = 1.11, FDR = 0.059). In addition, we identified a PRS association with a secondary trait besides cognitive disorders. We observed an inverse association of AD PRS with gouty arthropathy (OR = 0.90, FDR = 0.05). PRS PheWAS was also conducted in other ancestry samples with phenotypes of at least 50 case and control subjects each (Supplementary Tables 2B–E), but no significant associations were found.

**TABLE 3:** Significant PheWAS results of Alzheimer’s disease polygenic risk score in the full European ancestry sample (*N* = 19,934).*^a^*

Phecodes	Description	Group	N total	N Cases	N Controls	OR	Raw *P*-value	Adjusted *P*-value*^b^*
290.11	Alzheimer’s disease	Mental disorders	17,290	168	17,122	1.26	3.17E-05	0.015
292.2	Mild cognitive impairment	Mental disorders	17,470	359	17,111	1.18	1.81E-05	0.013
292.3	Memory loss	Mental disorders	18,583	1,470	17,113	1.10	2.88E-06	0.004
290.1	Dementias	Mental disorders	17,585	479	17,106	1.14	1.26E-04	0.046
274.11	Gouty arthropathy	Endocrine/metabolic	19,489	656	18,833	0.90	1.73E-04	0.050

*OR, odds ratio.*

*^a^All the values were based on results from multivariable logistic regression analyses in each sample, in which “no disease/symptom” was used as the reference group, and adjusted for age, sex, and first five PC sets.*

*^b^The adjusted p-value was calculated by controlling the FDR.*

### Determining Causality Between Alzheimer’s Disease and Secondary Phenotypes

To investigate whether the secondary association of gouty arthropathy and AD PRS was due to patients with both AD and gouty arthropathy, we reexamined the AD PRS-gout association after excluding AD cases. After adjusting for the same demographic variables (age, sex, number of follow-up years, and first five genetic PCs), the inverse association between AD PRS and gouty arthropathy was still significant (OR = 0.91, *p* = 0.01). We also evaluated the association between gouty arthropathy and AD phenotype in our European sample. Variables that influence both the exposure (gouty arthropathy) and outcome (AD) can cause a spurious association in observational studies (McNamee, 2005). We performed bivariate analyses to find factors that potentially confound the association between gouty arthropathy and AD (Supplementary Table 3). Hypertension, diabetes, stroke, and hyperlipid were significantly associated with both gouty arthropathy and AD. These were adjusted as confounders in subsequent models. Although there was a crude positive association between gouty arthropathy and AD (OR = 2.48, 95% CI: 1.11, 4.79), no significant association was found after adjustments of demographic and comorbidity variables mentioned above (Table 4).

**TABLE 4:** Results of logistic regression and one-sample Mendelian randomization testing associations and causality between gouty arthropathy and Alzheimer’s disease, European ancestry (*N* = 14,511).*^a^*

	Logistic regression test for association		One-sample MR test for causality	
	Odds ratio (95 CI%)		Beta coefficient*^d^*	*p*-value
Crude	2.48	(1.11, 4.79)	–29.71	0.04*
Adjusted demographics*^b^*	1.13	(0.49, 2.28)	–5.71	0.03*
Adjusted health conditions*^c^*	1.02	(0.44, 2.09)	–3.62	0.06

*AD, Alzheimer’s disease; CI, Confidence Interval; MR, Mendelian randomization; PRS, polygenic risk score.*

*^a^All the values were based on results from multivariable logistic regression analyses in each sample, in which “no Alzheimer’s disease” was used as the reference group.*

*^b^Adjusted for age, sex, number of follow-up years, and five ancestry-specific PC sets.*

*^c^Adjusted for history of hypertension, diabetes, stroke, and hyperlipid in addition to variables in b.*

^d^Beta coefficients for the causal estimates from gouty arthropathy to AD. *significant test statistics (p < 0.05).

Next, we examined whether a lower risk of AD was a consequence of gouty arthropathy with a one-sample MR framework (Supplementary Figure 1). A test of inferred causality of gouty arthropathy on AD was conducted using AD PRS as the instrumental variable since its association with gouty arthropathy was statistically significant (Table 3), which met the relevance assumption of MR. As shown in Table 4, the causality of gouty arthropathy on AD no longer held after adjusting for the same demographic and comorbidity variables mentioned above (one-sample MR with sequential probit models, *p* = 0.06). The results suggest that gouty arthropathy is not a causal protective factor of AD in our European ancestry sample.

We further tested whether gout is the causal protective risk factors for AD using a two-sample MR approach. This two-sample MR method does not directly test whether AD PRS is the instrumental variable, but rather uses multiple variants below a given *p*-value threshold from GWAS as the instrumental variable. We used the Kunkle et al. (2019) AD GWAS and the gout GWAS conducted by Tin et al. (2019) to perform two-sample MR analyses. Similar to our one-sample MR, no significant causal relationship was consistently found from gout to AD using multiple methods that include IVW, MR-Egger, weighted median, and weighted mode (Table 5). The F-statistic indicates adequate instrument strength (liberal: 86.76 > 10; conservative: 123.49 > 10). There was no heterogeneity across different methods or directional pleiotropy found using multiple measures (see “Subjects and Methods”). We also performed a sensitivity analysis to examine the reverse causality of AD on gout. Both one-sample and two-sample MR results showed no causal relationship between AD and gout (Supplementary Table 4).

**TABLE 5:** Two-sample Mendelian randomization to test causal relationship between gout status and Alzheimer’s disease.*^a^*

Instrument	Mendelian randomization method	Beta	SE	*P*-value	Sensitivity test	Results
Evaluate gout causal for AD						
Liberal	IVW (fixed effects)	0.022	0.019	0.24	F statistic (combined instrument)	86.76
*p* < 1E-06	IVW (multiplicative random effects)	0.022	0.020	0.25	Cochran’s Q (for IVW)	*p* = 0.34
(n_SNP = 48)	MR Egger	0.079	0.032	0.02*	MR-Egger intercept	*p* = 0.03
	Weighted median	0.057	0.029	0.05	MR-PRESSO global test	*p* = 0.26
	Weighted mode	0.054	0.028	0.06	I^2^ test	0.99
Conservative	IVW (fixed effects)	0.025	0.021	0.22	F statistic (combined instrument)	123.49
*p* < 5E-08	IVW (multiplicative random effects)	0.025	0.019	0.19	Cochran’s Q (for IVW)	*p* = 0.67
(n_SNP = 29)	MR Egger	0.059	0.034	0.10	MR-Egger intercept	*p* = 0.22
	Weighted median	0.054	0.030	0.07	MR-PRESSO global test	*p* = 0.20
	Weighted mode	0.056	0.030	0.08	I^2^ test	0.99

*IVW, inverse variance weighted; SE, standard error.*

^a^The F-statistics was not able to be calculated because the Kunkle GWAS summary statistics did not report allele frequency. *significant test statistics (p < 0.05).

## Discussion

Alzheimer’s disease is a complex disease determined by interactions between genetic risk factors and environmental modifiers (Baumgart et al., 2015; Xu et al., 2015; Fu et al., 2021; Ware et al., 2021). In our study, we conducted a comprehensive, ancestry specific PheWAS study using cumulative genetic risk of AD in a real-world academic medical center population. We provided evidence for the value of AD PRS to aid in identifying individuals who are genetically at risk of AD, and also other related phenotypes including MCI, memory loss and dementias in the European ancestry. We identified non-neurodegenerative diseases, especially gout, associated with AD PRS. Understanding horizontal pleiotropy for AD genetic risk is essential to broaden our understanding for the genetic architecture of AD. These PheWAS results also provide insights on potential side effects of drugs targeting these genetic risk factors in AD (Nguyen et al., 2019). For example, because AD PRS and gout have a negative association, drugs targeting AD genetic risk factors may increase risk of gout. Finally, we performed thorough analyses evaluating the causality between significant associations, which shows that gout was not a causal risk factor for AD. The evaluation of causality is an important component to infer the temporal order of diseases and better understand protective and risk factors of AD.

We constructed an AD PRS that summarized the aggregated AD genetic risks based on prior GWAS. We found moderate prediction power of the AD PRS in the European and East Asian ancestry sample, but poor predictive power in other non-European ancestry samples. We expected poor performance in non-European ancestry samples as our methods for computing the AD PRS depended on summary statistics from a GWAS including only participants of the European ancestry (Kunkle et al., 2019). Prior studies have found in multiple diseases that PRS constructed from European ancestry GWAS results in poor predictive performance in non-European ancestry populations (Duncan et al., 2019; Martin et al., 2019). Furthermore, we had small sample sizes for PheWAS in non-European ancestry samples (Table 2). The significant association of the European GWAS-based AD PRS with East Asian ancestry may be due to some shared genetic architecture for AD genetic risk. Prior studies found associations between a polygenic risk model using significant AD risk loci from European AD GWAS and AD in Chinese cohorts (Xiao et al., 2015; Zhou et al., 2020).

We then performed a primary PheWAS. In the European samples, we observed a significant positive association between AD PRS and AD, along with multiple cognitive phenotypes (MCI, memory loss, and dementias). Prior studies have identified an association of AD PRS with MCI (Logue et al., 2019) and the conversion of MCI to AD (Chaudhury et al., 2019; Logue et al., 2019). Our study further supports that AD PRS is associated with MCI in an EHR cohort. We also observed a borderline association between AD PRS and delirium dementia and amnestic and other cognitive disorders. Whereas it is known Alzheimer’s disease and dementias are risk factors for delirium (Fick et al., 2002; Fong et al., 2009), prior work has not evaluated the association of AD PRS and delirium. The association of AD PRS and these cognitive phenotypes including memory loss and dementias may also be due to the fact they are comorbid with or precede a diagnosis of Alzheimer’s disease (Varatharajah et al., 2019; Alzheimer’s Association, 2021). Our results from an EHR cohort suggest that using AD PRS to predict not only AD but also MCI and delirium should be further explored.

We also found a significant association between gouty arthropathy and AD PRS. We conducted multiple sensitivity analyses to explore whether the associations of AD PRS with gout and AD PRS with AD were driven by horizontal pleiotropy, in which genetic variants convey risk independently to two different phenotypes, or vertical pleiotropy, in which genetic variants convey risk to one phenotype, which in turn raises or lowers risk for the secondary phenotype (Zheutlin et al., 2019). In the European ancestry sample, we observed that gout was not causally related to AD using one-sample and two-sample MR. This result was also consistent with the null association found between gout and AD given by logistic regressions testing gout and AD without considering AD genetic risk and correcting for confounders (Table 4). Although gout commonly has an earlier age of onset than AD (Lu et al., 2016), which indicates that there is unlikely to be a causal relationship from AD to gout, we tested for reversal causality from AD to gout in our study as a sensitivity analysis and the null-causal relationship still held. Taken as a whole, gout is a horizontal pleiotropic factor of AD; that is, AD genetic variants have a negative effect on gout, but gout is not a causal risk factor for AD when considering other confounders.

The association of gout with AD has had mixed findings in prior work. Hyperuricemia is the key causal precursor for gout and has been proposed as a mechanistic link to AD (Lu et al., 2016). Uric acid is considered as a major natural antioxidant in plasma that reduces oxidative stress and protects against free radicals, which are elevated in AD (Tuppo and Forman, 2001; Polidori and Mecocci, 2002; Reddy, 2006; Al-Khateeb et al., 2015). Other cross-sectional studies of serum uric acid reported no difference in concentration in AD and MCI patients compared to healthy controls (Polidori and Mecocci, 2002).

There are several distinct aspects to our study. We used LDpred2 method to build our AD PRS. Since association tests in GWASs are typically performed one SNP at a time, the presence of strong correlation structures across the genome, also known as LD, will likely cause bias in the independent effect estimates (Choi et al., 2020). LDpred is a popular method for deriving polygenic scores to account for LD. It implements a Bayesian shrinkage model which uses a prior on effect sizes and LD information from an external reference panel to infer the posterior mean effect size of each SNP (Vilhjálmsson et al., 2015). LDpred2 is an updated version of LDpred that addresses the issues of model misspecification while improving the computational efficiency. Specifically, LDpred2 (auto model) allows the learning of the two LDpred parameters (the proportion of causal variants *p* and the SNP heritability h^2^) from data, which can therefore be applied to data without the need of a validation dataset to choose best-performing hyperparameters (Privé et al., 2020). In addition, we used Firth’s corrected logistic regression in our PheWAS analyses. The Firth’s bias correction can solve the problem of separation in logistic regression and provide well-controlled type I error rates for unbalanced case–control studies with relatively small sample counts (Wang, 2014). Finally, we used thorough MR analyses to study causal inferences of gout and AD. MR has the advantages of removing unmeasured confounding, and the use of both one-sample and two-sample MR could be complementary to each other. The advantage of one-sample MR is the use of individual participant data rather than summary data, whereas the advantage of two-sample MR is the increased statistical power and thus can provide more robust causal results. However, some assumptions, such as the exclusion restriction assumption, are difficult to completely verify as all true confounders for gout and AD are unknown (Burgess et al., 2015).

There are limitations to this study. Given the non-significant results of AD PRS and AD in non-European ancestry samples, the PheWAS results may not be generalizable to these populations. Although large AD GWAS is not currently available in other ancestry groups, future work should perform PheWAS with AD PRS from ancestry specific GWAS. Because thorough MR analysis did not identify a causal relationship between gout and AD, and MR methods do not consider temporal data, we did not consider the temporal ordering of gout and AD diagnosis.

In summary, this study expands our understanding of AD genetic and clinical risk factors and provides a framework for evaluating horizontal and vertical pleiotropy that can be used in aging research. With the growing number of real-world EHR linked with genetic data, continued research will improve our ability to use genetics, biomarkers, and clinical risk factors, some of which will be causal, for early disease prediction and treatment.

## Consortium/Group and Collaborative Authors

UCLA Precision Health Data Discovery Repository Working Group: Anna L. Antonio, Maryam Ariannejad, Angela M. Badillo, Brunilda Balliu, Yael Berkovich, Michael Broudy, Tony Dang, Chris Denny, Eleazar Eskin, Eran Halperin, Brian L. Hill, Ankur Jain, Vivek Katakwar, Clara Lajonchere, Clara Magyar, Sheila Minton, Ghouse Mohammed, Ariff Muhamed, Pabba Pavan, Michael A. Pfeffer, Nadav Rakocz, Akos Rudas, Rey Salonga, Timothy J. Sanders, Paul Tung, Vu Vu, and Ailsa Zheng. UCLA Precision Health ATLAS Working Group: Ruth Johnson, Yi Ding, Alec Chiu, Jae-Hoon Sul, Sriram Sankraraman, and Bogdan Pasaniuc.

## Data Availability Statement

The data analyzed in this study is subject to the following licenses/restrictions: individual electronic health record data are not publicly available due to patient confidentiality and security concerns. Requests to access these datasets should be directed to corresponding author.

## Ethics Statement

Ethical review and approval was not required for the study on human participants in accordance with the local legislation and institutional requirements. The patients/participants provided their written informed consent to participate in this study.

## Author Contributions

MF analyzed the data. MF and TC interpreted the data analysis and were major contributors in writing the manuscript. All authors read and approved the final manuscript.

## Conflict of Interest

The authors declare that the research was conducted in the absence of any commercial or financial relationships that could be construed as a potential conflict of interest.

## Publisher’s Note

All claims expressed in this article are solely those of the authors and do not necessarily represent those of their affiliated organizations, or those of the publisher, the editors and the reviewers. Any product that may be evaluated in this article, or claim that may be made by its manufacturer, is not guaranteed or endorsed by the publisher.
